# Effects of Dietary Fish Meal Replacement With Yellow Mealworm (*Tenebrio molitor*) Meal on Growth, Intestinal Microbiota, Hepatopancreas Metabolites, and Immune Defense Against DIV1 in Giant Freshwater Prawn (*Macrobrachium rosenbergii*)

**DOI:** 10.1155/anu/2904475

**Published:** 2026-07-20

**Authors:** Cui Liu, Qiusheng Jing, Jinbo Lu, Li Wang, Qincheng Huang, Yutong Zheng, Yukun Jie, Junjun Yan, Jilun Meng, Tiantian Ye, Zhimin Gu

**Affiliations:** ^1^ Xianghu Laboratory, Hangzhou 311231, Zhejiang, China; ^2^ Gansu Agricultural University, Lanzhou 730070, Gansu, China, gsau.edu.cn; ^3^ Zhejiang Wanli University, Ningbo, Zhejiang, 315100, China, zwu.edu.cn; ^4^ College of Life Science, Shaoxing University, Shaoxing 312000, Zhejiang, China, usx.edu.cn

**Keywords:** giant freshwater prawn, gut microbiota, immunity, metabolites, yellow mealworm meal

## Abstract

The yellow mealworm (*Tenebrio molitor*) stands out among insect protein sources for its ability to convert low‐value agricultural by‐products into valuable nutrients. This study evaluated the effects of replacing fish meal with yellow mealworm meal on growth performance, nutritional composition, intestinal microbiota, hepatopancreas metabolites, and immune defense against decapod iridescent virus 1 (DIV1) in giant freshwater prawn. Five isonitrogenous and isolipidic diets were formulated with yellow mealworm meal replacing fish meal at 0% (FM30), 10% (FM27), 20% (FM24), 40% (FM18), and 60% (FM12). A total of 750 prawns were randomly distributed into 15 tanks (three replicates per diet, 50 prawns per replicate) and cultured for 56 days, followed by a DIV1 challenge test. Results showed that Replacement of up to 60% of fish meal did not adversely affect growth performance, feed utilization, or the crude protein, crude lipid, and amino acid profiles in muscle. Muscle monounsaturated fatty acids (MUFAs) increased linearly with mealworm inclusion (*p* < 0.05). The FM12 group exhibited elevated arachidonic acid (ARA) and *n*−6 PUFA levels and reduced docosahexaenoic acid (DHA; *p* < 0.05), while the *n*−3/*n*−6 ratio remained unchanged. Gut microbiota composition shifted favorably, with increased abundance of Firmicutes and beneficial *Lactococcus* in the FM12 and FM18 groups (*p* < 0.05). Metabolic adaptation in the hepatopancreas involved glycerophospholipid metabolism, nucleotide metabolism, and pyruvate metabolism pathways. Following DIV1 challenge, the FM12 group showed significantly higher survival, increased plasma glutathione peroxidase (GPX) activity, decreased malondialdehyde (MDA) levels, and reduced hepatopancreatic apoptosis (*p* < 0.05). Immune‐related upregulation of *hpo*, *warts*, *mats*, and *ifn-α*, alongside downregulation of *caspase-3*, was also observed (*p* < 0.05). In conclusion, replacing 60% of dietary fish meal with yellow mealworm meal is a nutritionally safe and beneficial strategy for giant freshwater prawn, effectively maintaining growth performance and muscle composition, modulating gut microbiota, and enhancing immune defense against DIV1.

## 1. Introduction

The shrimp aquaculture industry has emerged as a leading force in global fishery production and is recognized as the second‐largest traded seafood worldwide, following salmon [1]. Freshwater prawns have attracted considerable interest due to their fast growth rate, resilience to diverse environmental conditions, high commercial value, and suitability for cultivation in low‐salinity brackish water environments [2]. The rapid expansion of the shrimp farming industry has substantially propelled the growth of the aquafeed sector [3]. Nevertheless, fish meal, which serves as the primary protein source in shrimp feed, is confronted with increasing challenges associated with resource unsustainability and soaring costs [4]. Therefore, integrating alternative protein sources into shrimp diets can effectively reduce dependence on fish meal and promote a more sustainable and cost‐effective feeding strategy [5].

Insects represent a promising and sustainable alternative to fish meal, offering a comparable nutritional profile rich in proteins, essential amino acids, and trace elements [6]. A pivotal development took place in 2017 when the European Union authorized the use of insect‐derived ingredients in aquafeeds, marking a transformative shift in aquaculture practices and catalyzing innovative research directions [7]. Among the insect species authorized under EU regulations, the yellow mealworm (*Tenebrio molitor*) stands out for its role in converting low‐value agricultural by‐products into a high‐value source of protein, amino acids, lipids, and various micronutrients [8]. Furthermore, several studies have demonstrated that partial replacement of fish meal with insect meal does not negatively affect the growth performance and health in farmed aquatic species [9–11].

Yellow mealworm powder is a nutrient‐rich ingredient, with a protein content ranging from 47% to 66% [12]. It is also enriched with various unsaturated fatty acids and an array of bioactive substances, including chitin and antimicrobial peptides [13]. Accordingly, dietary supplementation with this powder has been demonstrated to promote growth, improve meat quality, and enhance immune function in several farmed fish species, such as grass carp (*Ctenopharyngodon idellus*) [14], large yellow croaker (*Larimichthys crocea*) [15], mirror carp (*Cyprinus carpio* var. specularis) [16], and gilthead sea bream (*Sparus aurata*) [17]. In crustaceans, studies have demonstrated that partial replacement of fish meal with mealworm meal can improve growth performance, enhance immune‐related enzyme activities, and modulate intestinal microbiota [18, 19], while mealworm meal also exhibits high protein and essential amino acid digestibility (72%–86%) with methionine as the first limiting amino acid, and replacing up to 100% of fish meal did not adversely affect growth or survival in *Litopenaeus vannamei* [20]. More recently, feeding live yellow mealworms to giant freshwater prawn (*Macrobrachium rosenbergii*) as a partial (50%) or complete (100%) replacement for commercial feed was found to have no negative effect on growth performance, phenoloxidase activity, and nitrite stress tolerance [21].

The giant freshwater prawn is a high‐value species originating from Asia and has gained significant importance in global aquaculture owing to its excellent growth rate, distinctive taste, and robust ability to adapt to various environmental conditions [22]. Feng et al. [23] reported that *T. molitor* protein enhanced growth and bacterial disease resistance in giant freshwater prawn. However, that study used purified protein as a supplement rather than a fish meal replacement strategy, and antiviral effects were not examined. The present study utilized yellow mealworm meal to perform a comprehensive assessment of its impact on giant freshwater prawns, encompassing growth, muscle composition, gut microbiota, hepatopancreatic metabolism, and immune defense against decapod iridescent virus 1 (DIV1). The findings are expected to provide critical evidence supporting the practical application of yellow mealworm meal in the prawn farming industry, thereby promoting the sustainable development of aquaculture.

## 2. Materials and Methods

All experimental procedures involving animals were approved by the Laboratory Animal Welfare and Ethics Committee of the Zhejiang Academy of Agricultural Sciences (Approval Number ZAASLA2026020807).

### 2.1. Experiment Diets

Five experimental diets with isonitrogenous and isolipidic compositions were formulated using fish meal, yellow mealworm meal, soybean meal, chicken byproduct meal, and soy protein concentrate as protein sources, supplemented with fish oil and soybean oil as the primary lipid sources. The fish meal was sourced from Tianjin Jinhualiang Import & Export Trade Co., Ltd. (Tianjin, China), while the yellow mealworm meal was obtained from Xi’an Youlanda Biotechnology Co., Ltd. (Xi’an, China). All other ingredients were provided by Shanghai Fanyi Biotechnology Co., Ltd. (Shanghai, China). Proximate and amino acid compositions of fish meal and yellow mealworm meal are presented in Table 1, and their fatty acid profiles are provided in Table 2. The composition and formulation of the experimental diets are detailed in Table 3. The control group (FM30) contained no yellow mealworm meal, while the four experimental groups replaced 10% (FM27), 20% (FM24), 40% (FM18), and 60% (FM12) of fish meal with yellow mealworm meal, respectively. Diet preparation followed the procedure described by Liu et al. [24]. All ingredients were passed through a 40‐mesh sieve, uniformly blended, and processed into granules with a diameter of 2 mm using a lab‐scale pelletizer (SYSLG30‐IV). The obtained pellets were air‐dried and kept at 4°C prior to use. The fatty acid profile of five diets is presented in Table 4.

**Table 1:** The proximate and amino acid compositions of fishmeal and yellow mealworm meal (% dry matter).

Proximate composition and amino acids	Fish meal	Yellow mealworm meal
Moisture	7.4	5.5
Crude protein	66.7	48.33
Crude lipid	7.8	23.7
Ash	17.6	8.3
Thr	2.79	2.01
Val	3.01	2.26
Met	1.85	1.19
Ile	2.64	1.69
Leu	4.74	2.87
Phe	2.64	2.98
Lys	5.01	3.34
His	2.3	1.41
Arg	3.82	2.51
ΣEAA	28.8	20.26
Asp	5.77	4.74
Ser	2.54	1.89
Glu	8.49	6.78
Gly	3.93	1.97
Ala	4.18	2.74
Tyr	2.3	3.44
Pro	2.58	2.1
ΣNEAA	29.79	23.66

Abbreviations: EAA, essential amino acid; NEAA, nonessential amino acid.

**Table 2:** The fatty acids profile of fishmeal and yellow mealworm meal (% total fatty acids).

Fatty acids	Fish meal	Yellow mealworm meal
C12:0	0.06	0.12
C14:0	6.28	2.11
C15:0	0.71	0.30
C16:0	24.61	24.23
C17:0	1.80	0.27
C18:0	7.28	4.20
C20:0	0.33	0.27
C24:0	0.61	0.04
ΣSFA	41.68	31.55
C14:1*n*−5	0.18	0.14
C16:1*n*−7	6.86	9.78
C17:1*n*−7	0.91	0.17
C18:1*n*−9 (OA)	6.99	29.04
C20:1*n*−9	0.57	0.14
ΣMUFA	15.51	39.27
C18:2*n*−6 (LA)	1.43	25.04
C18:3*n*−6 (GLA)	0.20	0.09
C20:3*n*−6	0.26	0.03
C20:4*n*−6 (ARA)	2.15	0.64
Σ*n*−6PUFA	4.04	25.80
C18:3*n*−3	0.70	2.65
C20:5*n*−3 (EPA)	15.27	0.67
C22:6*n*−3 (DHA)	22.80	0.05
Σ*n*−3 PUFA	38.77	3.38

Abbreviations: AARA, arachidonic acid; DHA, docosahexaenoic acid; EPA, eicosapentaenoic acid; GLA, gamma‐linolenic acid; LA, linoleic acid; MUFA, monounsaturated fatty acids; OA, oleic acid; PUFA, polyunsaturated fatty acids; SFA, saturated fatty acids; Σ*n*−3 PUFA, *n*−3 polyunsaturated fatty acids; Σ*n*−6 PUFA, *n*−6 polyunsaturated fatty acids.

**Table 3:** Feed formulation and proximate composition (% dry matter).

Ingredients	FM30	FM27	FM24	FM18	FM12
Fish meal	30.0	27.0	24.0	18.0	12.0
Yellow mealworm meal	0.0	4.1	8.3	16.5	24.5
Soybean meal	15.0	15.0	15.0	15.0	15.0
Chicken byproduct meal	12.0	12.0	12.0	12.0	12.0
Soy protein concentrate	7.0	7.0	7.0	7.0	7.0
Fish oil	2.0	1.7	1.3	0.6	0.2
Soybean oil	2.0	1.7	1.3	0.6	0.2
Soybean lecithin	1.0	1.0	1.0	1.0	1.0
Wheat flour	14.0	14.0	14.0	14.0	14.0
Monocalcium Phosphate	1.0	1.0	1.0	1.0	1.0
Vitamin premix	2.0	2.0	2.0	2.0	2.0
Mineral premix	2.0	2.0	2.0	2.0	2.0
Cholesterol	0.5	0.5	0.5	0.5	0.5
Choline	0.5	0.5	0.5	0.5	0.5
Carboxymethyl‐cellulose (CMC)	3.0	3.0	3.0	3.0	3.0
Butylated hydroxytoluene (BHT)	0.1	0.1	0.1	0.1	0.1
L‐methionine	0.3	0.3	0.3	0.3	0.3
Microcrystalline cellulose	7.6	7.1	6.7	5.9	4.7
Total	100.0	100.0	100.0	100.0	100.0
Proximate composition (In dry matter)				—	
Crude protein (%)	40.8	40.9	41.0	40.7	41.1
Crude lipid (%)	9.0	9.4	9.4	9.8	10.8
Moisture (%)	7.2	6.8	7.1	7.6	6.6
Ash (%)	12.0	12.0	11.8	11.3	11.0
Crude fiber (%)	4.5	3.7	3.8	3.7	3.9
Gross energy (KJ/g)	19.61	19.78	19.88	20.10	20.34
Organic matter (%)	80.8	81.2	81.1	81.1	82.4
Neutral detergent fiber (%)	8.3	6.9	9.1	9.0	9.5
Acid detergent fiber (%)	4.1	4.9	4.2	4.5	5.9
Chitosan (%)	0.48	0.56	1.25	1.79	1.93

*Note:* Vitamin premix (per kg diet): vitamin A, 8000 IU; vitamin D_3_, 2000 IU; vitamin E, 100 mg; vitamin K_3_, 7.5 mg; vitamin B_1_, 15 mg; vitamin B_2_, 15 mg; vitamin B_6_, 12.5 mg; vitamin B_12_, 0.05 mg; D‐biotin, 0.25 mg; D‐calcium pantothenate, 40 mg; folic acid, 5 mg; niacinamide, 50 mg; vitamin C, 140 mg; inositol, 120 mg; ethoxyquin, 5 mg. Mineral premix (per kg diet): FeSO_4_, 40 mg; CuSO_4_·5 H_2_O, 25 mg; MnSO_4_·4 H_2_O, 10 mg; ZnSO_4_, 100 mg; MgSO_4_·7 H_2_O, 200 mg; CoCO_3_, 0.35 mg; KI, 0.05 mg; Na_2_SeO_3_, 0.3 mg.FM30, FM27, FM24, FM18, and FM12 denoted diets in which yellow mealworm meal replaced fishmeal at 0%, 10%, 20%, 40%, and 60%, respectively.

**Table 4:** The fatty acids profile of the experimental diets (% total fatty acids).

Fatty acids	FM30	FM27	FM24	FM18	FM12
C12:0	0.03	0.04	0.06	0.07	0.09
C14:0	1.83	1.87	1.97	1.97	1.95
C15:0	0.25	0.26	0.28	0.27	0.28
C16:0	19.43	20.35	21.46	23.24	24.13
C17:0	0.66	0.62	0.62	0.49	0.41
C18:0	6.46	6.25	6.22	5.87	5.62
C20:0	0.35	0.35	0.34	0.30	0.28
C24:0	0.44	0.43	0.39	0.34	0.29
ΣSFA	29.46	30.17	31.34	32.56	33.04
C14:1*n*−5	0.05	0.07	0.08	0.09	0.10
C16:1*n*−7	2.63	3.46	4.70	6.11	7.33
C17:1*n*−7	0.30	0.28	0.30	0.23	0.24
C18:1*n*−9 (OA)	21.03	21.86	22.56	24.54	26.03
C20:1*n*−9	0.46	0.42	0.39	0.27	0.25
ΣMUFA	24.48	26.08	28.03	31.25	33.95
C18:2*n*−6 (LA)	31.55	30.58	28.48	26.85	25.83
C18:3*n*−6 (GLA)	0.08	0.08	0.08	0.08	0.08
C20:3*n*−6	0.14	0.13	0.12	0.09	0.08
C20:4*n*−6 (ARA)	0.77	0.77	0.81	0.78	0.75
Σ*n*−6 PUFA	32.53	31.57	29.48	27.80	26.73
C18:3*n*−3	3.75	3.56	3.27	2.88	2.65
C20:5*n*−3 (EPA)	4.16	3.71	3.40	2.53	1.79
C22:6*n*−3 (DHA)	5.62	4.91	4.48	2.99	1.83
Σ*n*−3 PUFA	13.52	12.18	11.15	8.40	6.28

*Note:* FM30, FM27, FM24, FM18, and FM12 denoted diets in which yellow mealworm meal replaced fish meal at 0%, 10%, 20%, 40%, and 60%, respectively.

Abbreviations: ARA, arachidonic acid; DHA, docosahexaenoic acid; EPA, eicosapentaenoic acid; GLA, gamma‐linolenic acid; LA, linoleic acid; MUFA, monounsaturated fatty acids; OA, oleic acid; PUFA, polyunsaturated fatty acids; SFA, saturated fatty acids; Σ*n*−3 PUFA, *n*−3 polyunsaturated fatty acids; Σ*n*−6 PUFA, *n*−6 polyunsaturated fatty acids.

### 2.2. Prawns and Feeding Management

Healthy prawns were sourced from WuXing District, Zhejiang Province, and acclimated in 13 m^3^ square tanks for 2 weeks prior to the experiment. At the start of the feeding trial, the prawns were subjected to a 24‐h fasting period. The feeding experiment was conducted in an indoor aquaculture facility. A total of 750 healthy prawns with uniform size and an initial body weight of 0.22 ± 0.003 g were randomly assigned to five experimental groups. Each group included three replicate plastic tanks (300 L per tank), with 50 prawns stocked in each tank. During the experimental period, all tanks were continuously aerated to maintain dissolved oxygen concentrations above 7.0 mg L^−1^, with ammonia‐N levels kept below 0.05 mg L^−1^ and water temperatures maintained at 25 ± 2°C under natural photoperiod conditions. Prawns were fed twice daily to apparent satiation throughout the 56‐day trial period.

### 2.3. Sample Collections and DIV1 Challenge Assessment

At the end of the feeding experiment, all prawns were deprived of feed for 24 h. Prawns from each tank were collectively weighed, and two prawns per tank were individually weighed prior to hemolymph extraction. The collected hemolymph was immediately mixed with an equal volume of ice‐cold anticoagulant solution and then centrifuged at 4°C (10 min, 2000 × *g*). The resulting supernatant was carefully removed and preserved for subsequent analyses. Then, hepatopancreas were excised and weighed. Two additional prawns from each tank were selected for histological examination of the hepatopancreas and intestine. The hepatopancreas was collected for molecular analyses, and intestinal microbiota samples were obtained. All sampling steps were performed on ice.

The DIV1 crude solution preparation and challenge test were according to the methods described by Liu et al. [24]. In brief, gill tissues collected from DIV1‐positive prawns were homogenized in PBS on ice. The homogenate was centrifuged at 3000 r/min for 20 min at 4°C, and the resulting supernatant was further centrifuged at 8000 r/min for 25 min at 4°C. The final supernatant was filtered through a 0.22 μm membrane and stored at 4°C as the viral stock. Based on preliminary trials, the semi‐lethal concentration was determined to be 1.95 × 10^7^ copies/mL, and 50 μL of this preparation was injected into each prawn. Plasma and hepatopancreas samples were collected for analysis at 24 h post‐DIV1 injection, while the remaining prawns were monitored to determine the cumulative survival rate over a 120 h period following the challenge.

### 2.4. Proximate Composition and Antioxidant Indicators Analysis

The proximate composition of feed and muscle samples was analyzed following the standard methods described by the AOAC [25]. The moisture content was determined by drying samples in an oven at 105°C until a constant weight was achieved, as specified in AOAC Method 930.15. Crude protein was measured using the Kjeldahl method with a Kjeltec 2300 analyzer (Foss Tecator, Sweden) in accordance with AOAC Method 984.13. The crude lipid content was assessed via ether extraction using a Soxtec System HT6 (Tecator Ltd., Haganas, Sweden), based on AOAC Method 954.02. The crude fiber content was evaluated using the ceramic fiber filter technique according to AOAC Method 962.09, while ash content was determined by combusting samples in a muffle furnace at 550°C for 12 h, following AOAC Method 942.05. Organic matter was calculated as the difference between dry matter and ash content. Gross energy was measured using a bomb calorimeter (Parr 1341, Parr Instrument Company, Moline, IL, USA) in line with GB/T 45104‐2024 [26]. Neutral detergent fibers and acid detergent fibers were analyzed according to GB/T 20806‐2022 [27] and NY/T 1459‐2022 [28], respectively. Amino acid composition was analyzed using an A300 amino acid analyzer (MembraPure, Hennigsdorf, Germany), and fatty acid profiles were determined by gas chromatography–mass spectrometry (GC–MS, Agilent 7890 A, Agilent Technologies, USA), following the procedures outlined by Liu et al. [29]. Chitosan detection was performed in compliance with GB/T 38479‐2021 [30].

Plasma levels of superoxide dismutase (SOD), glutathione peroxidase (GPX), and malondialdehyde (MDA) were quantified using assay kits sourced from Nanjing Jiancheng Bioengineering Institute (Nanjing, China) [29].

### 2.5. Intestinal Microbiome Analysis

DNA extraction and PCR‐based amplification of intestinal microbiota were conducted following the protocol described by Liu et al. [24]. Equimolar quantities of the resulting amplicons were combined and subjected to paired‐end sequencing using the Illumina MiSeq system (Illumina, San Diego, USA) at Shanghai Majorbio Biopharm Technology Co., Ltd., in accordance with standard operating procedures. Sequence data analysis was performed on the Majorbio Cloud Platform (www.majorbio.com). The original sequencing reads have been deposited into the NCBI under the BioProject accession number PRJNA1399547.

### 2.6. Hepatopancreas Metabolome Analysis

For metabolomic analysis, the procedures for LC‐MS/MS detection, data preprocessing, and identification of differential metabolites were carried out following the protocol outlined by Wang et al. [31]. The raw metabolite dataset has been archived in OMIX, hosted by the China National Center for Bioinformation and the Beijing Institute of Genomics, Chinese Academy of Sciences, and is accessible under the Accession Number OMIX014237.

### 2.7. Histology and Tunel Staining

The hepatopancreas and intestine tissues were processed for fixation, paraffin embedding, sectioning, and hematoxylin and eosin (HE) staining according to the protocol outlined by Liu et al. [24]. For the detection of apoptosis in the hepatopancreas, TUNEL assays were conducted following the method described by Hadeed et al. [32], with subsequent imaging and data analysis. ImageJ Launcher was utilized for quantitative analysis [33].

### 2.8. Quantitative Real‐Time PCR

Total RNA was extracted, and cDNA was synthesized following the manufacturer’s guidelines (TransGen Biotech, Beijing, China). The integrity of the isolated RNA was assessed via 1% agarose gel electrophoresis. Quantitative real‐time PCR with SYBR Green dye was performed on the QuantStudio 3 platform (Applied Biosystems, Thermo Fisher Scientific Inc., USA) using the PerfectStart Green qPCR SuperMix (TransGen Biotech). The reference genes used in this study were 18 S. The primer sequences and qPCR efficiency are listed in Table S1. Melt curve analysis was performed to confirm a single specific product for each primer pair. Gene expression levels were normalized and calculated using the 2^−∆∆CT^ method [34].

### 2.9. Statistical Analysis

All statistical analyses were performed at the tank level using the mean value per tank as the experimental unit. Before performing any statistical analyses, normality and homogeneity of variances were assessed, and one‐way ANOVA was used for data analysis [35]. Significant differences in mean values across treatment groups were identified using Tukey’s multiple range test, with statistical significance set at *p*  < 0.05 [36]. To evaluate the impact of varying levels of yellow mealworm meal substitution on all measured variables and parameters, linear and quadratic polynomial regression analyses were conducted [35]. Furthermore, the Kaplan–Meier survival analysis, combined with the log‐rank *χ* ^2^ test, was employed to compare the cumulative survival rates of prawns infected with DIV1 among the different treatment groups [24]. Data are presented as the mean ± SEM.

## 3. Results

### 3.1. Growth Performance

There was no significant difference in the specific growth rate, weight gain rate, feed utilization, and hepatosomatic index of prawns when fish meal was substituted with yellow mealworm meal (Table 5, *p*  > 0.05).

**Table 5:** The growth performance of *M. rosenbergii* fed the experiment diets.

Indexes	Groups					*p*‐Value		
	FM30	FM27	FM24	FM18	FM12	ANOVA	Linear	Quadratic
IBW (g)	0.22 ± 0.002	0.22 ± 0.001	0.22 ± 0.004	0.22 ± 0.000	0.22 ± 0.001	NA	NA	NA
FBW (g)	2.22 ± 0.04	2.19 ± 0.08	2.20 ± 0.06	2.15 ± 0.01	2.23 ± 0.02	0.849	0.413	0.491
SGR (%/day)	4.12 ± 0.01	4.09 ± 0.05	4.10 ± 0.02	4.05 ± 0.02	4.13 ± 0.03	0.829	0.266	0.078
WGR (%)	902.98 ± 5.18	886.31 ± 29.77	891.68 ± 10.93	867.18 ± 9.73	912.4 ± 14.19	0.415	0.188	0.073
FCR	2.16 ± 0.21	2.02 ± 0.07	2.29 ± 0.17	2.31 ± 0.08	2.1 ± 0.12	0.875	0.807	0.526
HSI (%)	4.00 ± 0.32	3.62 ± 0.33	3.74 ± 0.26	3.81 ± 0.26	4.02 ± 0.31	0.402	0.566	0.336

*Note:* SGR, specific growth rate (%/day) = 100 × [ln (final body weight) − ln (initial body weight)]/days. WGR, weight gain rate (%) = 100% × (final body weight − initial body weight)/initial body weight. FCR, feed conversion ratio = feed intake/(final body weight − initial body weight). HSI (hepatosomatic index, %) = hepatopancreas weight/whole body weight × 100.FM30, FM27, FM24, FM18, and FM12 denoted diets in which yellow mealworm meal replaced fishmeal at 0%, 10%, 20%, 40%, and 60%, respectively.

Abbreviations: FBW (g): final body weight, IBW (g): initial body weight; NA, not applicable.

### 3.2. Amino Acids and Fatty Acids Profile of Muscle

The crude protein, crude lipid, and amino acid composition in the muscle of prawns showed no significant differences among groups fed varying levels of yellow mealworm meal (Table 6, *p*  > 0.05). However, muscle fatty acid profiles were notably affected by dietary treatments (Table 7). Monounsaturated fatty acids (MUFAs) increased linearly with increasing levels of yellow mealworm meal (*p*  < 0.05). Arachidonic acid (ARA) and *n*−6 polyunsaturated fatty acids (PUFAs) were significantly higher in the FM12 group (*p*  < 0.05). In contrast, eicosapentaenoic acid (EPA), *n*−3 PUFAs, and total PUFAs remained unchanged across diets, whereas docosahexaenoic acid (DHA) was significantly reduced in the FM12 group (*p*  < 0.05), no significant difference was observed between the FM18 group and the other dietary groups.

**Table 6:** The proximate and amino acid compositions in muscle of *M. rosenbergii* fed different levels of yellow mealworm meal (% dry matter).

Indexes	Groups					*p*‐Value		
	FM30	FM27	FM24	FM18	FM12	ANOVA	Linear	Quadratic
Crude protein	20.07 ± 0.41	20.9 ± 0.50	21.7 ± 0.66	20.67 ± 0.23	20.33 ± 0.27	0.169	0.881	0.362
Crude lipid	1.27 ± 0.12	1.27 ± 0.09	1.23 ± 0.15	1.23 ± 0.03	1.10 ± 0.06	0.737	0.183	0.365
Thr	0.80 ± 0.02	0.78 ± 0.03	0.85 ± 0.02	0.78 ± 0.03	0.78 ± 0.01	0.210	0.542	0.711
Val	0.92 ± 0.02	0.91 ± 0.04	0.97 ± 0.03	0.90 ± 0.03	0.89 ± 0.01	0.339	0.488	0.617
Met	0.54 ± 0.03	0.46 ± 0.06	0.52 ± 0.09	0.48 ± 0.04	0.56 ± 0.02	0.678	0.619	0.508
Ile	0.87 ± 0.02	0.89 ± 0.03	0.95 ± 0.03	0.87 ± 0.02	0.86 ± 0.01	0.182	0.700	0.561
Leu	1.51 ± 0.04	1.56 ± 0.06	1.64 ± 0.04	1.53 ± 0.03	1.49 ± 0.03	0.173	0.595	0.351
Phe	0.86 ± 0.01	0.85 ± 0.04	0.90 ± 0.02	0.84 ± 0.03	0.78 ± 0.05	0.274	0.190	0.161
His	0.38 ± 0.01	0.37 ± 0.02	0.38 ± 0.02	0.35 ± 0.02	0.35 ± 0.00	0.632	0.081	0.296
Lys	1.71 ± 0.03	1.76 ± 0.09	1.85 ± 0.04	1.72 ± 0.04	1.66 ± 0.04	0.198	0.449	0.325
Arg	2.06 ± 0.03	2.03 ± 0.05	2.17 ± 0.06	2.02 ± 0.03	2.02 ± 0.05	0.227	0.549	0.731
EAA	10.09 ± 0.51	9.60 ± 0.42	10.23 ± 0.27	9.49 ± 0.19	9.82 ± 0.31	0.569	0.558	0.829
Asp	2.21 ± 0.04	2.20 ± 0.12	2.34 ± 0.06	2.21 ± 0.07	2.11 ± 0.06	0.369	0.372	0.320
Ser	0.82 ± 0.01	0.81 ± 0.03	0.84 ± 0.03	0.81 ± 0.02	0.80 ± 0.01	0.606	0.410	0.619
Glu	3.31 ± 0.04	3.32 ± 0.17	3.49 ± 0.08	3.31 ± 0.10	3.23 ± 0.05	0.490	0.458	0.392
Gly	1.41 ± 0.05	1.41 ± 0.07	1.35 ± 0.08	1.42 ± 0.05	1.33 ± 0.05	0.775	0.370	0.707
Ala	1.19 ± 0.03	1.23 ± 0.04	1.26 ± 0.02	1.21 ± 0.01	1.16 ± 0.02	0.122	0.377	0.091
Tyr	0.72 ± 0.02	0.71 ± 0.03	0.74 ± 0.01	0.71 ± 0.00	0.72 ± 0.02	0.766	0.825	0.964
Pro	0.70 ± 0.01	0.68 ± 0.03	0.79 ± 0.07	0.73 ± 0.06	0.70 ± 0.09	0.638	0.956	0.611
NEAA	10.37 ± 0.17	10.34 ± 0.34	10.81 ± 0.24	10.39 ± 0.19	9.81 ± 0.17	0.122	0.273	0.151

*Note:* FM30, FM27, FM24, FM18, and FM12 denoted diets in which yellow mealworm meal replaced fishmeal at 0%, 10%, 20%, 40%, and 60%, respectively.

Abbreviations: EAA, essential amino acid; NEAA, nonessential amino acid.

**Table 7:** The fatty acids profile in muscle of *M. rosenbergii* fed different levels of yellow mealworm meal (% total fatty acids).

Indexes	Groups					*p*‐Value		
	FM30	FM27	FM24	FM18	FM12	ANOVA	Linear	Quadratic
C14:0	1.13 ± 0.00	1.06 ± 0.05	1.09 ± 0.03	1.08 ± 0.04	1.00 ± 0.02	0.161	0.102	0.317
C16:0	25.87 ± 0.28	25.95 ± 0.08	25.87 ± 0.02	26.36 ± 0.29	25.78 ± 0.35	0.506	0.831	0.513
C18:0	12.24 ± 0.02^c^	12.36 ± 0.22^c^	12.08 ± 0.39^bc^	11.29 ± 0.2^a^	11.44 ± 0.11^ab^	0.022	0.003	0.012
SFA	39.24 ± 0.30	39.37 ± 0.26	39.03 ± 0.39	38.73 ± 0.16	38.22 ± 0.43	0.170	0.010	0.035
C16:1*n*−7	1.13 ± 0.14^ab^	0.97 ± 0.02^a^	1.36 ± 0.06^bc^	1.52 ± 0.04^b^	1.51 ± 0.09^b^	0.002	0.001	0.005
C18:1*n*−9 (OA)	13.97 ± 1.71^a^	16.51 ± 0.17^b^	17.98 ± 0.37^bc^	18.15 ± 0.28^bc^	18.83 ± 0.52^c^	0.004	0.006	0.012
MUFA	14.96 ± 1.68^a^	17.48 ± 0.18^b^	19.35 ± 0.33^bc^	19.67 ± 0.31^c^	20.34 ± 0.56^c^	0.002	0.003	0.007
C18:2*n*−6 (LA)	19.08 ± 0.03	18.41 ± 0.08	18.70 ± 0.28	18.45 ± 0.37	19.19 ± 0.16	0.153	0.416	0.088
C20:2*n*−6	0.76 ± 0.01^c^	0.80 ± 0.04^c^	0.75 ± 0.02^bc^	0.68 ± 0.01^ab^	0.67 ± 0.01^a^	0.019	0.002	0.011
C20:4*n*−6 (ARA)	3.13 ± 0.16^a^	3.21 ± 0.09^a^	3.16 ± 0.06^a^	3.71 ± 0.05^a^	4.60 ± 0.44^b^	0.003	<0.001	<0.001
Σ*n*−6 PUFA	23.08 ± 0.22^a^	22.42 ± 0.03^a^	22.62 ± 0.23^a^	22.83 ± 0.35^a^	24.23 ± 0.12^b^	0.002	0.006	<0.001
C18:3*n*−3	1.25 ± 0.03	1.20 ± 0.04	1.22 ± 0.03	1.20 ± 0.05	1.14 ± 0.04	0.413	0.075	0.187
C20:5*n*−3 (EPA)	11.29 ± 0.71	11.83 ± 0.38	10.93 ± 0.56	10.81 ± 0.33	10.69 ± 0.95	0.711	0.246	0.523
C22:5*n*−3	0.82 ± 0.05^b^	0.86 ± 0.01^b^	0.76 ± 0.05^b^	0.76 ± 0.00^b^	0.63 ± 0.05^a^	0.023	0.004	0.012
C22:6*n*−3 (DHA)	6.58 ± 0.55^b^	6.85 ± 0.06^b^	6.10 ± 0.35^b^	5.99 ± 0.22^ab^	4.96 ± 0.25^a^	0.012	0.001	0.004
Σ*n*−3PUFA	19.52 ± 1.24	20.74 ± 0.32	19.00 ± 0.89	18.76 ± 0.50	17.20 ± 0.94	0.130	0.049	0.200
PUFA	42.79 ± 1.91	43.15 ± 0.30	41.62 ± 0.71	41.59 ± 0.16	41.44 ± 0.97	0.480	0.123	0.269
Σ*n*−3/Σ*n*−6	0.82 ± 0.1	0.89 ± 0.02	0.81 ± 0.05	0.79 ± 0.03	0.69 ± 0.05	0.130	0.016	0.042

*Note:* Values in the same row with different superscript letters are significantly different (*p* < 0.05). FM30, FM27, FM24, FM18, and FM12 denoted diets in which yellow mealworm meal replaced fishmeal at 0%, 10%, 20%, 40%, and 60%, respectively.

Abbreviations: ARA, arachidonic acid; DHA, docosahexaenoic acid; EPA, eicosapentaenoic acid; GLA, gamma‐linolenic acid; LA, linoleic acid; MUFA, monounsaturated fatty acids; OA, oleic acid; PUFA, polyunsaturated fatty acids; SFA, saturated fatty acids; Σ*n*−3 PUFA, *n*−3 polyunsaturated fatty acids; Σ*n*−6 PUFA, *n*−6 polyunsaturated fatty acids.

### 3.3. Histological Analysis of the Hepatopancreas and Intestine

**Figure 1:**
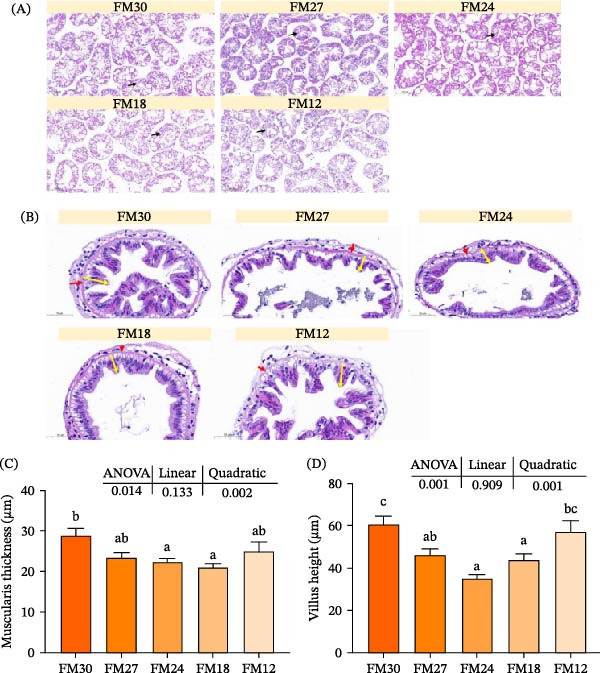
The hepatopancreas (A) and intestinal (B) morphology and histological parameters of intestine (C, D) of *M. rosenbergii* fed different levels of yellow mealworm meal. Black arrows mark goblet cells (A); red arrows mark muscularis thickness and yellow arrows mark villous height (B). Bars with different lowercase letters mean significant changes among groups after DIV1 injection (*p*  < 0.05). FM30, FM27, FM24, FM18, and FM12 denoted diets in which yellow mealworm meal replaced fish meal at 0%, 10%, 20%, 40%, and 60%, respectively.

The hepatopancreatic structure was not affected by the diets (Figure 1A), whereas intestinal morphology was significantly altered in prawns fed different levels of yellow mealworm meal (Figure 1B). The thickness of the intestinal muscularis was significantly reduced in the FM24 and FM18 groups (Figure 1C, *p*  < 0.05), and villus height was also significantly decreased in the yellow mealworm meal supplementation groups (Figure 1D, *p*  < 0.05).

### 3.4. Intestinal Microbiota Composition

**Figure 2:**
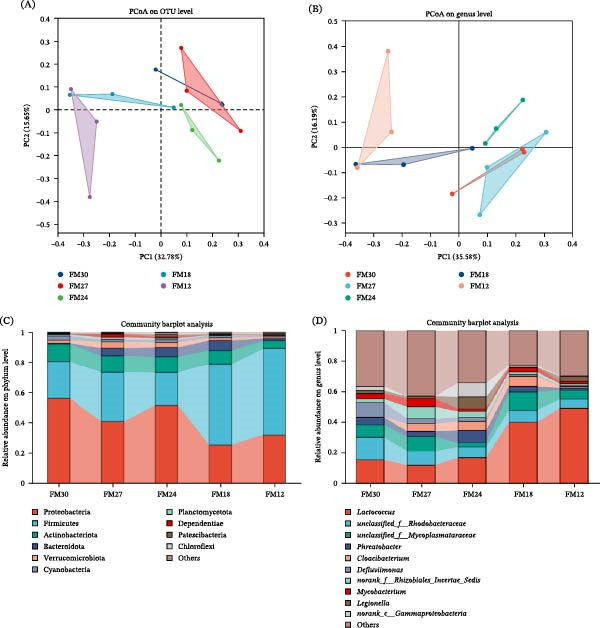
PCoA on OTU (A) and genus levels (B), intestinal microbiota distribution at the phylum (C) and genus (D) level in *M. rosenbergii* fed different levels of yellow mealworm meal. FM30, FM27, FM24, FM18, and FM12 denoted diets in which yellow mealworm meal replaced fish meal at 0%, 10%, 20%, 40%, and 60%, respectively.

The sequence coverage obtained from high‐throughput sequencing of intestinal microbiota 16S rRNA exceeded 90%, with no significant differences observed among the experimental groups (Table S2). Alpha diversity indices, including Sobs, Shannon, Ace, and Chao, remained unchanged following the inclusion of yellow mealworm meal in the diet (Table S2, *p*  > 0.05). The PCoA analysis results revealed that, apart from the FM27 group, the other three yellow mealworm meal supplementation groups were clearly separated from the FM30 group at both the OTU and genus levels (Figure 2A,B). Regarding intestinal microbiota composition, the dominant phyla included Proteobacteria, Firmicutes, Actinobacteriota, and Bacteroidota (Figure 2C), while the predominant genera were *Lactococcus*, unclassified *Rhodobacteraceae*, unclassified *Mycoplasmataceae*, *Phreatobacter*, and *Cloacibacterium* (Figure 2D). The abundance of Firmicutes was significantly higher in the FM12 group (Table 8, *p*  < 0.05), whereas Bacteroidota abundance increased only in the FM24 and FM18 groups (Table 8, *p*  < 0.05). The abundance of *Lactococcus* showed a linear increase with dietary yellow mealworm meal supplementation (Table 8, *p*  < 0.001). *Cloacibacterium* was significantly enriched in the FM27, FM24, and FM18 groups (Table 8, *p*  < 0.05), while *Legionella* levels increased specifically in the FM24 group (Table 8, *p*  < 0.05).

**Table 8:** The main Phylum and Genus levels in intestinal microflora of *M. rosenbergii* fed different levels of yellow mealworm meal.

Indexes	Groups											*p*‐Value			
	FM30		FM27		FM24		FM18		FM12			ANOVA		Linear	Quadratic
Phylum levels															
Proteobacteria	56.27 ± 6.13		41.18 ± 5.85		51.68 ± 8.41		25.31 ± 7.06		32.44 ± 12.82			0.126		0.036	0.091
Firmicutes	24.51 ± 6.49^ab^		32.10 ± 1.48^abc^		21.54 ± 4.00^a^		53.76 ± 13.09^bc^		56.95 ± 12.98^c^			0.049		0.006	0.026
Actinobacteriota	11.54 ± 1.44		11.24 ± 2.35		10.73 ± 2.89		8.93 ± 1.49		5.14 ± 1.77			0.238		0.017	0.048
Bacteroidota	0.40 ± 0.23^a^		5.04 ± 0.78^abc^		6.05 ± 1.27^bc^		6.56 ± 2.87^bc^		1.36 ± 0.07^c^			0.040		0.914	0.005
Verrucomicrobiota	2.06 ± 0.59		4.10 ± 1.87		3.33 ± 0.66		1.81 ± 1.25		1.12 ± 0.35			0.360		0.169	0.245
Cyanobacteria	2.69 ± 0.24		1.58 ± 0.04		1.77 ± 0.25		1.38 ± 0.73		0.68 ± 0.40			0.061		0.005	0.022
Genus levels															
* Lactococcus*	15.62 ± 1.22^a^		12.33 ± 4.32^a^		17.24 ± 2.66^a^		40.35 ± 8.45^b^		49.49 ± 8.60^b^			0.003		<0.001	0.001
* Phreatobacter*	4.82 ± 2.37		3.49 ± 1.89		8.17 ± 4.99		3.70 ± 1.6		1.26 ± 0.67			0.520		0.309	0.375
* Cloacibacterium*	0.21 ± 0.06^a^		5.02 ± 0.78^bc^		5.87 ± 1.24^bc^		6.52 ± 2.88^c^		1.27 ± 0.05^ab^			0.037		0.893	0.005
* Defluviimonas*	1.46 ± 0.96		3.34 ± 1.71		2.60 ± 0.75		1.08 ± 0.46		0.44 ± 0.32			0.286		0.125	0.193
* Mycobacterium*	3.52 ± 1.31		5.30 ± 1.78		1.64 ± 0.28		2.96 ± 1.46		1.57 ± 0.51			0.246		0.155	0.379
* Legionella*	1.97 ± 0.31^a^		1.46 ± 0.28^a^		7.79 ± 2.65^b^		0.39 ± 0.11^a^		5.29 ± 2.03^ab^			0.030		0.542	0.830

*Note:* Values in the same row with different superscript letters are significantly different (*p* < 0.05). FM30, FM27, FM24, FM18, and FM12 denoted diets in which yellow mealworm meal replaced fishmeal at 0%, 10%, 20%, 40%, and 60%, respectively.

### 3.5. Metabolomic Profiling of Hepatopancreas

**Figure 3:**
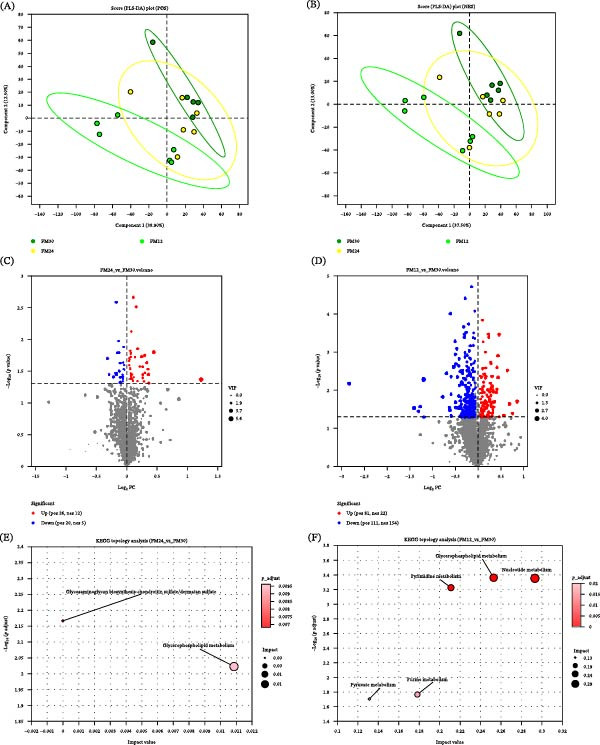
PLS‐DA (A, B), Vocano plot (C, D), and KEGG topology analysis (E, F) of differential metabolites in the hepatopancreas of *M. rosenbergii*. FM30, FM24, and FM12 denoted diets in which yellow mealworm meal replaced fishmeal at 0%, 20%, and 60%, respectively.

The PLS‐DA results revealed a clear distinction in the metabolic profiles of the hepatopancreas between FM30 and FM12 groups (Figure 3A,B). There were 38 metabolites with significant upregulation and 25 metabolites with downregulation in the FM24 group (Figure 3C), while there were 103 metabolites with significant upregulation and 265 metabolites with down‐regulation (Figure 3D). The differentially expressed metabolites were significantly enriched in the Glycosaminoglycan biosynthesis and Glycerophospholipid metabolism KEGG pathways in the FM24 vs. FM30 groups (*Q* <0.05, Figure 3E), and the differentially expressed metabolites were significantly enriched in the Nucleotide metabolism, Glycerophospholipid metabolism, pyrimidine metabolism, purine metabolism, and pyruvate metabolism KEGG pathways in the FM12 vs. FM30 groups (*Q* <0.05, Figure 3F).

### 3.6. The Cumulative Survival Rate of Prawns Following DIV1 Injection

**Figure 4:**
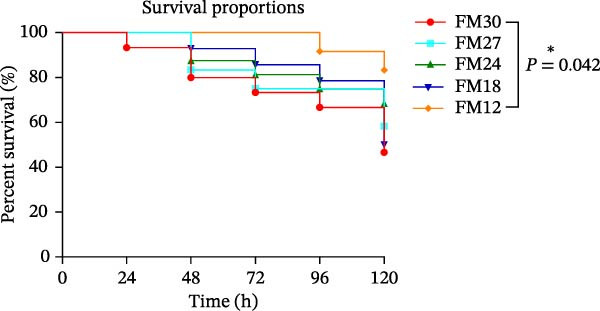
The cumulative survival rate of *M. rosenbergii* fed with different levels of yellow mealworm meal following DIV1 infection. Asterisk ( ^∗^) represent *p*  < 0.05. FM30, FM27, FM24, FM18, and FM12 denoted diets in which yellow mealworm meal replaced fish meal at 0%, 10%, 20%, 40%, and 60%, respectively.

The cumulative survival rates of prawns in the FM30, FM27, FM24, FM18, and FM12 groups after 120 h of DIV1 infection were 46.67%, 58.33%, 68.75%, 50.00%, and 83.33%, respectively (Figure 4). Notably, the FM12 group exhibited a significantly higher survival rate compared to the FM30 group (*p* < 0.05).

### 3.7. Plasma Antioxidant Enzyme and Tunel Staining

**Figure 5:**
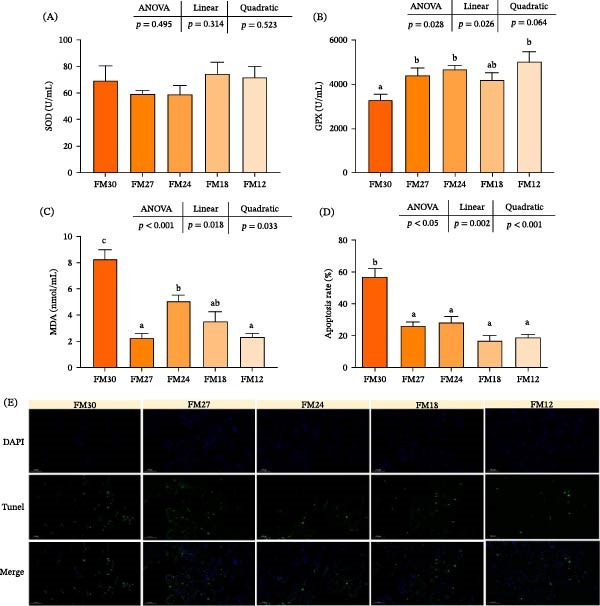
The plasma antioxidant parameters and Tunel staining in the hepatopancreas of *M. rosenbergii* fed with different levels of yellow mealworm meal following DIVl infection. (A) SOD: superoxide dismutase; (B) GPX: glutathione peroxidase; (C) MDA: malondialdehyde; (D) apoptosis rate of hepatopancreas; (E) Tunel staining of the hepatopancreas. Bars with different lowercase letters mean significant changes among groups after DIV1 injection (*p* < 0.05). FM30, FM27, FM24, FM18, and FM12 denoted diets in which yellow mealworm meal replaced fishmeal at 0%, 10%, 20%, 40%, and 60%, respectively.

No significant differences were detected in the activity of the antioxidant enzyme SOD in prawn plasma among all groups following DIV1 injection (Figure 5A). Nevertheless, the GPX activity was notably higher in the groups supplemented with yellow mealworm meal (Figure 5B, *p*  < 0.05). Moreover, these groups showed lower MDA levels (Figure 5C, *p*  < 0.05).

TUNEL staining of the hepatopancreas is shown in Figure 5E, revealing a significantly decreased apoptosis rate in the yellow mealworm meal‐supplemented groups following DIV1 injection (Figure 5D, *p*  < 0.05).

### 3.8. Hippo Signaling Pathway and Immune‐Related Genes Expression

**Figure 6:**
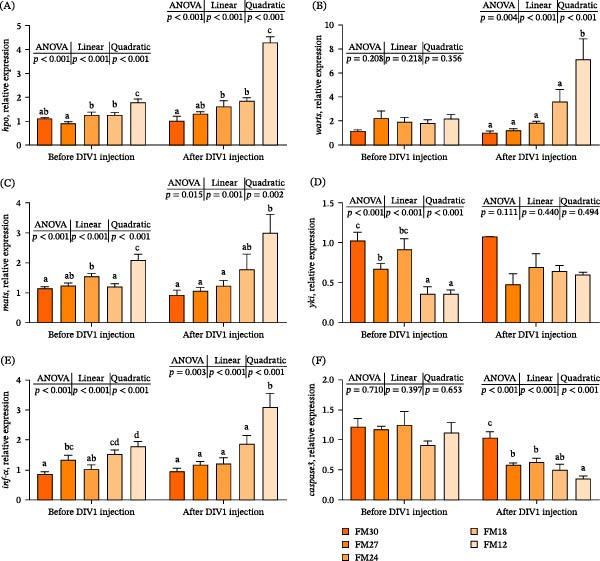
Expression levels of Hippo signaling pathway and immune‐related genes of *M. rosenbergii* fed with different levels of yellow mealworm meal before and after DIV1 injection. (A) *hpo*: serine/threonine‐protein kinase hippo; (B) *warts*: serine/threonine‐protein kinase warts‐like; (C) *mats*: mob kinase activator‐like 1; (D) *yki*: transcriptional coactivator yorkie; (E) *ifn-α*: *Interferon-α*; (F) *caspase3*. Bars with different lowercase letters mean significant changes among groups (*p* < 0.05). FM30, FM27, FM24, FM18, and FM12 denoted diets in which yellow mealworm meal replaced fishmeal at 0%, 10%, 20%, 40%, and 60%, respectively..

Since the differentially genes before and after DIV1 infection were both significantly enriched in the Hippo signaling pathway, we analyzed the expression of key genes in hepatopancreas involved in this signaling pathway (Figure 6). Prior to DIV1 injection, the relative expression levels of *hpo* and *mats* were significantly upregulated in the FM12 group (*p* < 0.05), while *yki* expression was significantly downregulated in both the FM18 and FM12 groups (*p* < 0.001). Additionally, *ifn-α* expression was significantly increased in groups supplemented with yellow mealworm meal (*p* < 0.001). Following DIV1 injection, the expression of *hpo*, *warts*, *mats*, and *ifn-α* was markedly upregulated in the FM12 group (*p* < 0.05), whereas caspase3 expression was significantly downregulated in the yellow mealworm meal supplementation groups (*p* < 0.001).

## 4. Discussion

In the present study, substitution of 60% dietary fish meal with yellow mealworm meal did not adversely affect the growth performance or feed utilization. These findings are consistent with previous researches on *L. vannamei* [20] and rainbow trout (*Oncorhynchus mykiss*) [37], where complete replacement of fish meal with yellow mealworm meal was achieved without negative impacts. Furthermore, growth enhancement has been observed in *L. vannamei* at replacement levels of up to 30% [18, 19], supporting the feasibility of partial inclusion. While it has been suggested that chitin in yellow mealworm could impair the digestibility of dry matter and protein [20], more recent studies indicated that insect flours with chitin and its derivatives may function as prebiotic and antibacterial agents, potentially improving growth and immune responses in crustaceans and fish [38, 39]. Notably, a prior study by Feng et al. [23] examined the effects of supplementing a fish meal‐free commercial aquafeed with purified yellow mealworm protein in giant freshwater prawn. Their findings indicated that a 12% inclusion level significantly enhanced growth performance, whereas increasing the supplementation to 16% yielded no additional benefits. In contrast, our study employs yellow mealworm meal as a direct substitute for fish meal. Given that giant freshwater prawns are an omnivorous species with a high tolerance for dietary protein [40], this may explain why replacing up to 60% of fish meal in our experiment did not adversely affect growth outcomes. Therefore, yellow mealworm meal represents a promising alternative protein source to fish meal in formulated diets for giant freshwater prawns, with no detrimental effects on growth performance under the tested replacement level.

The amino acid composition in the muscle of giant freshwater prawns remained stable across all dietary treatments, indicating that the replacement of fish meal with yellow mealworm meal did not disrupt normal amino acid homeostasis or protein synthesis. Similar findings regarding muscle crude protein and crude lipid have been reported in *L. vannamei* and Atlantic salmon (*Salmo salar*) [41, 42]. Research on the effects of yellow mealworm meal on the amino acid composition of shrimp muscle remains limited. In contrast, studies in fish have shown variable results. For instance, Zhang et al. (2023) reported that increasing dietary inclusion of this meal significantly elevated the muscle contents of methionine, glycine, tyrosine, and proline (expressed as % dry matter), while total essential, nonessential, and total amino acids remained unchanged [15]. However, Qu et al. (2025) only observed no significant changes in total essential and flavor amino acid pools without providing specific data on individual amino acids [43].

Yellow mealworm meal inclusion significantly altered the fatty acid profile in the muscle of giant freshwater prawn, corresponding to the trends observed in the dietary fatty acid composition. The yellow mealworm meal contained substantially higher levels of MUFAs, particularly oleic acid (OA, C18:1*n*−9), significantly increased in the yellow mealworm meal, which was 4.1‐fold greater than that in fish meal. Similarly, total n‐6 PUFAs (Σ*n*−6 PUFAs) were significantly elevated in yellow mealworm meal, with linoleic acid (LA, C18:2*n*−6) reaching 17.5 times the concentration found in fish meal. Despite this pronounced dietary increase, muscle LA levels did not change significantly. In contrast, a significant increase in ARA (C20:4*n*−6) was detected in prawn muscle from the 60% fish meal replacement group. Yellow mealworm meal is notably deficient in the long‐chain *n*−3 PUFAs EPA (C20:5*n*−3) and DHA (C22:6*n*−3). leading to a decrease in dietary EPA and DHA content with increasing replacement levels. While muscle EPA content remained unchanged across all groups, DHA levels decreased significantly only in the 60% replacement group. This pattern suggests the prawn’s selective retention of physiologically essential long‐chain PUFAs, specifically ARA, EPA, and DHA, suggesting its potential ability to elongate and desaturate precursor fatty acids, such as LA, into these longer‐chain derivatives [42, 44]. In this study, the *n*−3/*n*−6 ratio ranged from 0.80 to 0.69, higher than results reported for yellow mealworm in *L. vannamei* study [42] but more aligned with findings in fish feeding study [45] and within the recommended range 0.25–1.0 for human intake [46].

The digestion and absorption of nutrients are closely associated with the intestinal absorptive area and structural integrity [47]. It has been hypothesized that chitin in yellow mealworm meal might cause damage to the intestinal structure of aquatic animals as insufficient endogenous chitinase allows undigested chitin to bind to digestive enzymes, thereby impairing nutrient digestion and absorption [14, 48]. The present study demonstrated that replacing fish meal with yellow mealworm meal reduced muscularis thickness and villus height, thereby diminishing the absorptive surface area. Interestingly, no significant difference was observed between the 60% fish meal replacement group and the control group. This phenomenon can potentially be ascribed to the capacity of chitin to enhance the abundance of intestinal microbiota that possess the capability to degrade chitin [49, 50]. Consistent with the findings of the study on *Hermetia illucens* meal in giant freshwater prawns [3], the yellow mealworm meal had no significant impact on the hepatopancreatic structure of the prawn.

The gut microbiota plays a significant role in influencing the physiological, nutritional, immunological, and metabolic processes of the host organism [51]. In the present study, substituting fish meal with yellow mealworm powder was observed to be associated with alterations in the gut microbiota composition. Although no marked differences in alpha diversity were observed across groups, PCoA analysis revealed distinct shifts in the microbial community structure, particularly in the 40% and 60% fish meal replacement groups. In these groups, the abundances of Firmicutes and Bacteroidota increased notably. Previous studies have reported that the ratio of Firmicutes to Bacteroidota is positively correlated with growth performance and antioxidant capacity in aquatic species [14], suggesting a potential link between the observed compositional shifts and the host physiological status. At the genus level, a notable increase in the relative abundance of *Lactococcus*, a widely recognized probiotic and fermentative bacterium, was detected at replacement levels of 20%, 40%, and 60%. *Lactococcus* has been previously shown to contribute to intestinal health in aquatic organisms like giant freshwater prawn through the production of advantageous secondary metabolites, which have been associated with improve growth performance and intestinal function [3, 52]. However, it should be noted that our compositional data do not directly demonstrate the functional activity of this genus, and further functional analyses would be required to confirm such effects. Conversely, the abundance of *Cloacibacterium*, a genus associated with potential disease transmission, decreased significantly at the 60% replacement level [53].

Previous studies have reported that dietary supplementation with yellow mealworm powder or its derivatives can enhance resistance against pathogenic bacteria and improve survival rates in fish and crustaceans following infection [23, 54, 55]. Furthermore, the present study demonstrated that yellow mealworm powder supplementation could improve the antiviral capacity in crustaceans. The primary findings include an increased survival rate after DIV1 infection, which is accompanied by enhanced plasma antioxidant activity, reduced levels of lipid peroxidation products, and attenuated hepatopancreas cell apoptosis in the 60% fish meal replacement group. These beneficial effects are likely attributable to the rich content of chitin and antimicrobial peptides in yellow mealworm powder [8, 56, 57]. Notably, a previous study has also reported that supplementation of a fish meal‑free commercial diet with extracted *T. molitor* proteins similarly conferred significantly increased resistance against bacterial pathogens, further supporting the immunostimulatory role of *T. molitor*‑derived products in giant freshwater prawns [23]. This also suggests that yellow mealworm meal may be able to replace high levels of fish meal, or even 100%, but this warrants further investigation under our experimental conditions.

Metabolomic profiling of the hepatopancreas further demonstrated that differential metabolites were mainly enriched in several key pathways, including glycerophospholipids metabolism, nucleotide metabolism, and pyruvate metabolism. Glycerophospholipids, as the most abundant membrane lipids, play a vital role in maintaining the membrane structure and physical properties [58]. The remodeling of glycerophospholipid metabolism likely supports normal cellular membrane function under altered dietary conditions. Furthermore, nucleotide metabolism is crucial for cellular energy balance [59], and pyruvate metabolism, a key pathway in glycolysis and oxidative phosphorylation, is crucial for energy homeostasis, mitochondrial quality control, and inflammation response [60]. When 60% of fish meal is replaced with yellow mealworm meal, giant freshwater prawns may reprogram the metabolic network in the hepatopancreas, particularly reconfiguring the energy production pathway, to compensate for potential changes in energy utilization efficiency caused by the unconventional protein sources.

The Hippo signaling pathway was traditionally recognized as a conserved regulator of cell growth, proliferation, and apoptosis [61], and recent studies have revealed its critical role in the immune response of shrimp to pathogens such as *Vibrio* and white spot syndrome virus (WSSV) [62, 63]. In this study, replacement of 20%, 40%, and 60% of fish meal with yellow mealworm meal significantly upregulated the expression of the *hpo* gene. Moreover, at the 60% replacement level, the expressions of *warts* and *mats* were also markedly increased. Concurrently, the expression of the antiviral cytokine *ifn-α* was elevated, while the pro‐apoptotic gene *caspase3* was significantly downregulated. These results may suggest that yellow mealworm meal activates the Hippo signaling pathway, positively regulates interferon production, and suppresses unnecessary apoptosis to enhance DIV1 defense.

## 5. Conclusions

This study demonstrated that yellow mealworm meal can effectively replace up to 60% of fish meal in the diets of giant freshwater prawns without compromising growth performance, feed utilization, or muscle proximate composition. Notably, the inclusion of yellow mealworm meal significantly modified the muscle fatty acid profile and favorably reshaped the gut microbiota, particularly by increasing the abundance of beneficial probiotics. Dietary inclusion of yellow mealworm meal, especially at 60% substitution, significantly improved resistance to DIV1 by activating the Hippo signaling pathway, upregulating *ifn-α* expression, and suppressing caspase‐3 expression. Metabolomics analysis further revealed that the prawns adapt through energy metabolic reprogramming to meet new nutritional demands and maintain physiological homeostasis. In conclusion, replacing 60% of dietary fish meal with yellow mealworm meal is a nutritionally safe and beneficial strategy for giant freshwater prawns, effectively maintaining growth performance and muscle composition, modulating gut microbiota, and enhancing immune defense against DIV1. However, given that the present study only examined replacement levels up to 60%, further research is first needed to evaluate the effects of yellow mealworm meal in diets with higher or complete fish meal replacement to determine the maximum feasible inclusion level for this species. In addition, large‑scale trials under commercial farming conditions and a comprehensive assessment framework encompassing nutritional, health, economic, and environmental outcomes should be prioritized in future studies.

## Author Contributions

**Cui Liu**: writing – original draft, visualization, methodology, investigation, funding acquisition. **Qiusheng Jing, Jinbo Lu, Yutong Zheng, and Yukun Jie:** methodology. **Li Wang and Qincheng Huang**: conceptualization. **Junjun Yan, Jilun Meng, and Tiantian Ye**: visualization. **Zhimin Gu**: writing – review and editing, supervision, funding acquisition.

## Funding

This study was funded by the Zhejiang Key Research and Development Program (Grant 2024SSYS0101), the National Key Research and Development Program of China (Grant 2024YFD2402400), and the National Natural Science Foundation of China (32202958).

## Conflicts of Interest

The authors declare no conflicts of interest.

## Supporting Information

Additional supporting information can be found online in the Supporting Information section.

## Supporting information


**Supporting Information** Table S1: Primer sequences of genes in quantitative real‐time PCR. Table S2: Alpha diversity of intestinal flora of *M. rosenbergii* fed different levels of yellow mealworm meal.

## Data Availability

All data generated or analyzed during this study are available from the corresponding author upon reasonable request.
