# Supplementary material for: Effects of Dietary Fish Meal Replacement With Yellow Mealworm (Tenebrio molitor) Meal on Growth, Intestinal Microbiota, Hepatopancreas Metabolites, and Immune Defense Against DIV1 in Giant Freshwater Prawn (Macrobrachium rosenbergii)
Source: Aquac Nutr. 2026 Jul 20;2026:2904475. doi: 10.1155/anu/2904475 (PMC13383005; doi:10.1155/anu/2904475)
Supplement: Supplementary file 1 — Supporting Information Table S1: Primer sequences of genes in quantitative real‐time PCR. Table S2: Alpha diversity of intestinal flora of M. rosenbergii fed different levels of yellow mealworm meal. [file ANU-2026-2904475-s001.docx]

**Table S1** Primer sequences of genes in quantitative real-time PCR.

| Gene | Primer sequence (5′-3′) | Accession number/Reference | PCR efficiency |
| --- | --- | --- | --- |
| *hpo* | F:GAGTCGCACAACAAACACAG | XM_ 067091919.1 | 1.90 |
|  | R: TACACTGCCATAGGACCCTT |  |  |
| *warts* | F:GGCCAACCACCATTTTTAGC | XM_067089470.1 | 2.01 |
|  | R:CCTAGCCGTTGTTCAGGATG |  |  |
| *mats* | F:GGGTGACACTGATTCCCTTC | XM_067096836.1 | 2.04 |
|  | R:TGAAAGTCTTGCTGGATCGG |  |  |
| *yki* | F: CTTCAGGCCAGACAACTCAG | XM_067132388.1 | 1.97 |
|  | R: CTCCCGTAGACTCATCCGTA |  |  |
| *ifn-α* | F:TACTCTCTTGACAGGTTCCCC | XM_067132446.1 | 1.93 |
|  | R: AGCTAGTACGAAGGGTGCT |  |  |
| *caspase3* | F: CGGATTCAAACGCGATGACC | Zhan et al., 2023* | 1.99 |
|  | R: GACGACAACGTGGTCTGACT |  |  |
| *18S* | F: TGTTACGGGTGACGGA | GQ131934.1 | 1.98 |
|  | R: AATTACGCAGACTCGGAAGA |  |  |

*hpo*: serine/threonine-protein kinase hippo; *warts*: serine/threonine-protein kinase warts-like;

*mats*: mob kinase activator-like 1; *yki*: transcriptional coactivator yorkie; *ifn-α*: Interferon-α.

* Zhan F, Zhou S, Shi F, Li Q, Lin L, Qin Z. Transcriptome analysis of *Macrobrachium rosenbergii* hemocytes in response to Staphylococcus aureus infection. Fish & Shellfish Immunology 2023; 139: 108927.

**Table S2** Alpha diversity of intestinal flora of *M. rosenbergii* fed different levels of yellow mealworm meal.

| **Indexes** | **Groups** | | | | |  | | ***P*-value** | | |
| --- | --- | --- | --- | --- | --- | --- | --- | --- | --- | --- |
|  | **FM30** | **FM27** | **FM24** | **FM18** | **FM12** | |  | **ANOVA** | **Linear** | **Quadratic** |
| Sobs | 511.75 ± 36.01 | 514 ± 101.00 | 605.25 ± 45.24 | 473.25 ± 68.38 | 419.75 ± 79.82 | |  | 0.06 | 0.233 | 0.305 |
| Shannon | 3.12 ± 0.47 | 2.95 ± 0.83 | 3.50 ± 0.24 | 2.76 ± 0.74 | 2.41 ± 0.58 | |  | 0.20 | 0.16 | 0.285 |
| Simpson | 0.12 ± 0.06 | 0.18 ± 0.19 | 0.09 ± 0.02 | 0.18 ± 0.12 | 0.25 ± 0.11 | |  | 0.24 | 0.139 | 0.295 |
| Ace | 602.21 ± 38.36 | 630.28 ± 126.47 | 721.7 ± 44.22 | 579.11 ± 70.57 | 505.27 ± 81.65 | |  | 0.06 | 0.254 | 0.241 |
| Chao | 580.5 ± 42.08 | 611.99 ± 116.54 | 712.4 ± 35.88 | 560.81 ± 76.96 | 495.8 ± 89.81 | |  | 0.06 | 0.304 | 0.293 |
| Coverage, % | 99.81 ± 0.02 | 99.78 ± 0.05 | 99.76 ± 0.02 | 99.79 ± 0.02 | 99.83 ± 0.02 | |  | 0.06 | 0.38 | 0.134 |

FM30, FM27, FM24, FM18, and FM12 denoted diets in which yellow mealworm meal replaced fishmeal at 0%, 10%, 20%, 40%, and 60%, respectively.
